# A six-year retrospective study on the causative agents of onychomycosis in China: the emergence of dematiaceous fungi

**DOI:** 10.3389/fmicb.2025.1582147

**Published:** 2025-05-01

**Authors:** Xin Ye, Jun Tian, Wanqing Liao, Weihua Pan, Zhe Liu, Jiaming Zhang, Li Yang, Lei Zhang

**Affiliations:** ^1^Department of Laboratory Medicine, The First Affiliated Hospital of Xi’an Jiao Tong University, Xi’an, China; ^2^Department of Dermatology, Shaanxi Provincial People’s Hospital, The Third Affiliated Hospital of Xi’an Jiao Tong University, Xi’an, China; ^3^Department of Dermatology, Shanghai Changzheng Hospital, Navy Medical University, Shanghai, China

**Keywords:** onychomycosis, epidemiology, *Trichophyton*, dematiaceous, dermatophytes

## Abstract

**Introduction:**

Onychomycosis, a common nail disease, is caused by a diverse range of pathogens worldwide. However, the epidemiology and pathogen profile of onychomycosis in China remain insufficiently characterized. This study aimed to investigate these aspects in a large Chinese hospital.

**Methods:**

A six-year retrospective analysis was conducted at a tertiary hospital in China, where nail samples from 298 patients who were clinically suspected of onychomycosis were cultured and analyzed to identify causative agents and clinical features.

**Results:**

Of the 298 samples, 51.00% (152) were positive for fungal infection. Young adults (18–30 years) comprised the majority of the patients, with a man-to-woman ratio of 1:1.45. Dermatophytes were the most prevalent causative agents (36.18%), followed by yeasts (28.29%) and non-dermatophyte molds (NDMs) (28.29%). Among dermatophytes, *Trichophyton* species (34.9%) were the most frequently identified, followed by *Candida* (21.7%) and dematiaceous fungi (8.6%). Dermatophytes were the predominant pathogens in the patients aged 18–50 years. The toenails (63.04%) were more commonly affected than the fingernails (36.96%), with bilateral toenail involvement (34.07%) being the most frequent.

**Conclusion:**

While dermatophytes remain the leading cause of onychomycosis in China, non-dermatophyte molds, particularly dematiaceous fungi, are emerging as significant pathogens. These organisms present unique treatment challenges and warrant increased clinical attention.

## 1 Introduction

Onychomycosis is the most common fungal infection of the nail, accounting for approximately 90% of toenail infections globally (Gupta et al., 2020; Verrier et al., 2012). The causative organisms of onychomycosis vary across the world and could be broadly classified into three categories: dermatophytes, non-dermatophyte molds (NDMs), and yeast-like fungi (Kardjeva et al., 2006). Among these, *Trichophyton rubrum* and *Candida* are commonly identified in dermatophytes and yeast-like fungi, respectively (Ghannoum and Isham, 2014). In general, dermatophytes remain the predominant causative agents of onychomycosis, with new fungi, including dematiaceous fungi, emerging (Gupta et al., 2021). The emerging threat of antifungal resistance highlights the importance of accurate diagnosis and susceptibility tests (Fisher et al., 2022).

Shaanxi Province is located in the northwest of China, characterized by a distinct monsoon climate and diverse latitudinal geography (Jing et al., 2023). Shaanxi Provincial People’s Hospital serves a regional population exceeding 100 million, with an annual outpatient volume of nearly two million. In this study, the pattern of causative agents at this hospital over the past 6 years (2017–2022) was investigated. Clinical and demographic features (age and gender) and affected sites (toenails/fingernails) were also analyzed. This study may serve as a call for attention to the emergence of NDMs (for instance, dematiaceous fungi) in onychomycosis, especially in long-neglected northwest China.

## 2 Materials and methods

### 2.1 Participants

All outpatients suspected of onychomycosis at Shaanxi Provincial People’s Hospital who had samples collected for fungal culture between 2017 and 2022 were identified. Among them, the clinical features (age, gender, causative pathogen, and affected toenails/fingernails) of the culture-positive samples were reviewed and recorded by two colleagues. When diagnosing the pathogens of onychomycosis, we followed the diagnostic criteria of the Chinese Onychomycosis Diagnosis and Treatment Guidelines (2015 Edition) (Dermatologist Branch of Chinese Medical Association, 2015). Specifically, the culture medium was placed at 25–28°C for 2–3 weeks. If the culture result identified dermatophytes, a diagnosis of onychomycosis was established. However, if yeasts or other molds exhibited growth of the same species at 6 out of 10 inoculation points, with corresponding hyphal or spore morphological features observed using direct microscopy, they were diagnosed as pathogenic fungi. The use of these criteria helped exclude the possibility of contamination.

For fungal culture, samples were inoculated onto two Sabouraud dextrose agar plates—one containing chloramphenicol and the other supplemented with chloramphenicol and cycloheximide. The plates were incubated at 26°C and 35°C for 4 weeks, followed by mass spectrometry identification and lactophenol cotton blue staining microscopy.

The present study complied with the ethical standards of the Helsinki Declaration II and was approved by the Medical Research Ethic Committee of Shaanxi Provincial People’s Hospital, Xi’an Jiaotong University (No. 2021-180). This study was solely observational, and a waiver of informed consent was approved by the Medical Research Ethic Committee of Shaanxi Provincial People’s Hospital, Xi’an Jiaotong University.

### 2.2 Research procedure

The present study analyzed the following aspects: (1) gender and age distribution among culture-positive patients suspected of onychomycosis; (2) fungi profiles in culture-positive patients suspected of onychomycosis; (3) predominant causative agents in different gender and age groups; and (4) distribution of causative agents in toenails and fingernails.

### 2.3 Statistical analysis

This study aimed to investigate the pathogen spectrum and clinical features of patients suspected of onychomycosis. Therefore, only descriptive methods were applied in this study. Sums and percentages were calculated and are presented in this research. GraphPad Prism 8 was used for figure preparation in this study.

## 3 Results

### 3.1 Study population

A total of 4,99,344 outpatient records from the Department of Dermatology were reviewed over 6 years, of which 3,458 were clinically suspected of onychomycosis. Among these, 298 samples suspected of onychomycosis were sent for fungal culture identification, with 152 (51.00%) culture-positive results. The demographic features of the patients are shown in Supplementary Table 1.

### 3.2 Gender and age distribution among all fungal culture-positive patients suspected of onychomycosis

**Figure 1:**
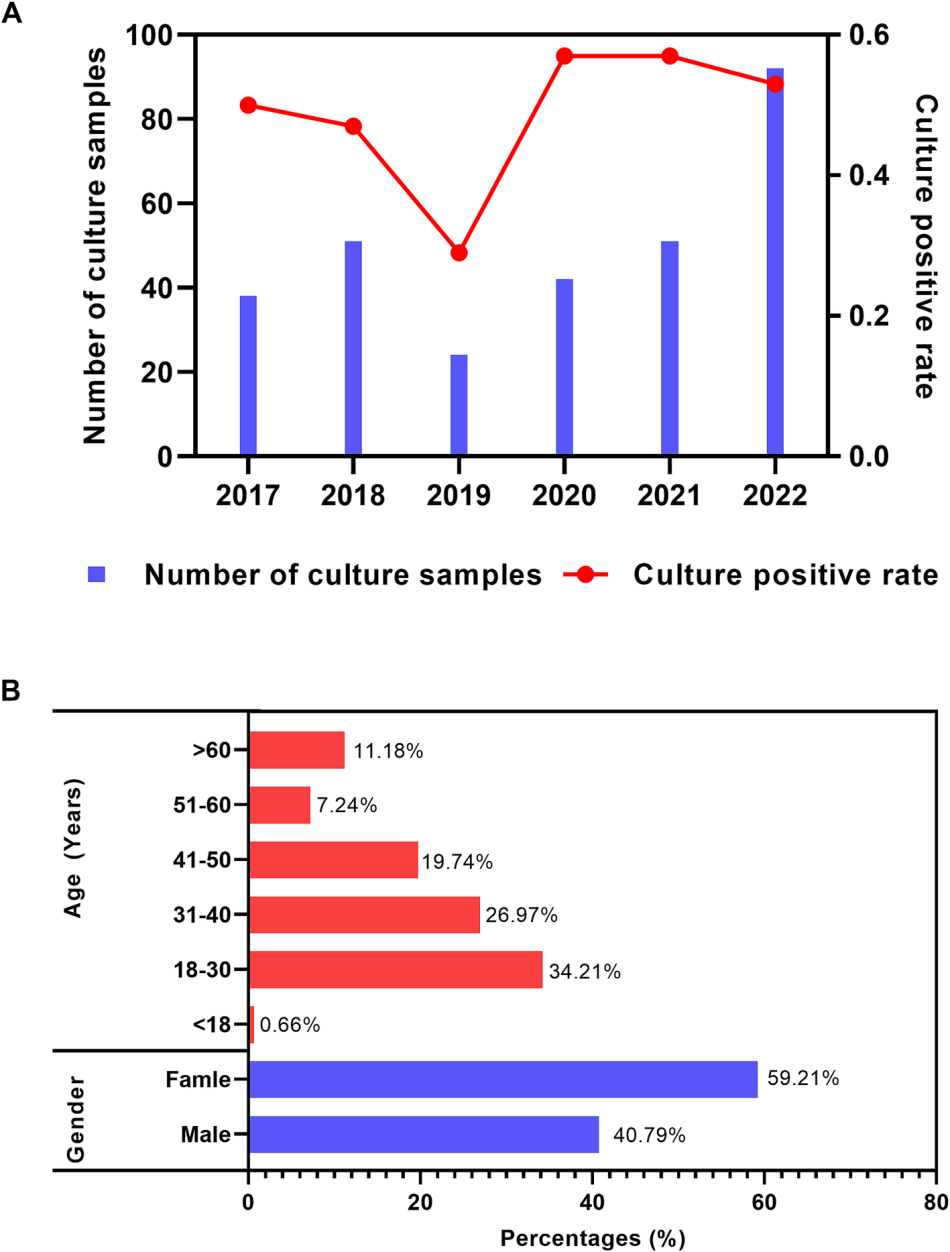
Distribution features of the fungal culture-positive patients suspected of onychomycosis. **(A)** Annual number of samples sent for fungal culture and corresponding culture-positive distribution. **(B)** Gender and age distribution among all fungal culture-positive patients suspected of onychomycosis.

During the past 6 years (2017–2022), the annual number of the samples sent for fungal culture increased from 38 (2017) to 92 (2022); meanwhile, the annual positive rate of the fungal culture samples was approximately 80%, except for 50% in 2019, as shown in Figure 1A. Regarding the distribution of the culture-positive samples across different age and gender groups, young adults (between 18 ~ 30 years) had the highest proportion (34.21%). The culture-positive rate demonstrated a decreasing trend with increasing age. The man-to-woman ratio was 1:1.45 (62:90), as shown in Figure 1B.

### 3.3 Non-dermatophyte molds emerged as the main causative agents of onychomycosis

**Figure 2:**
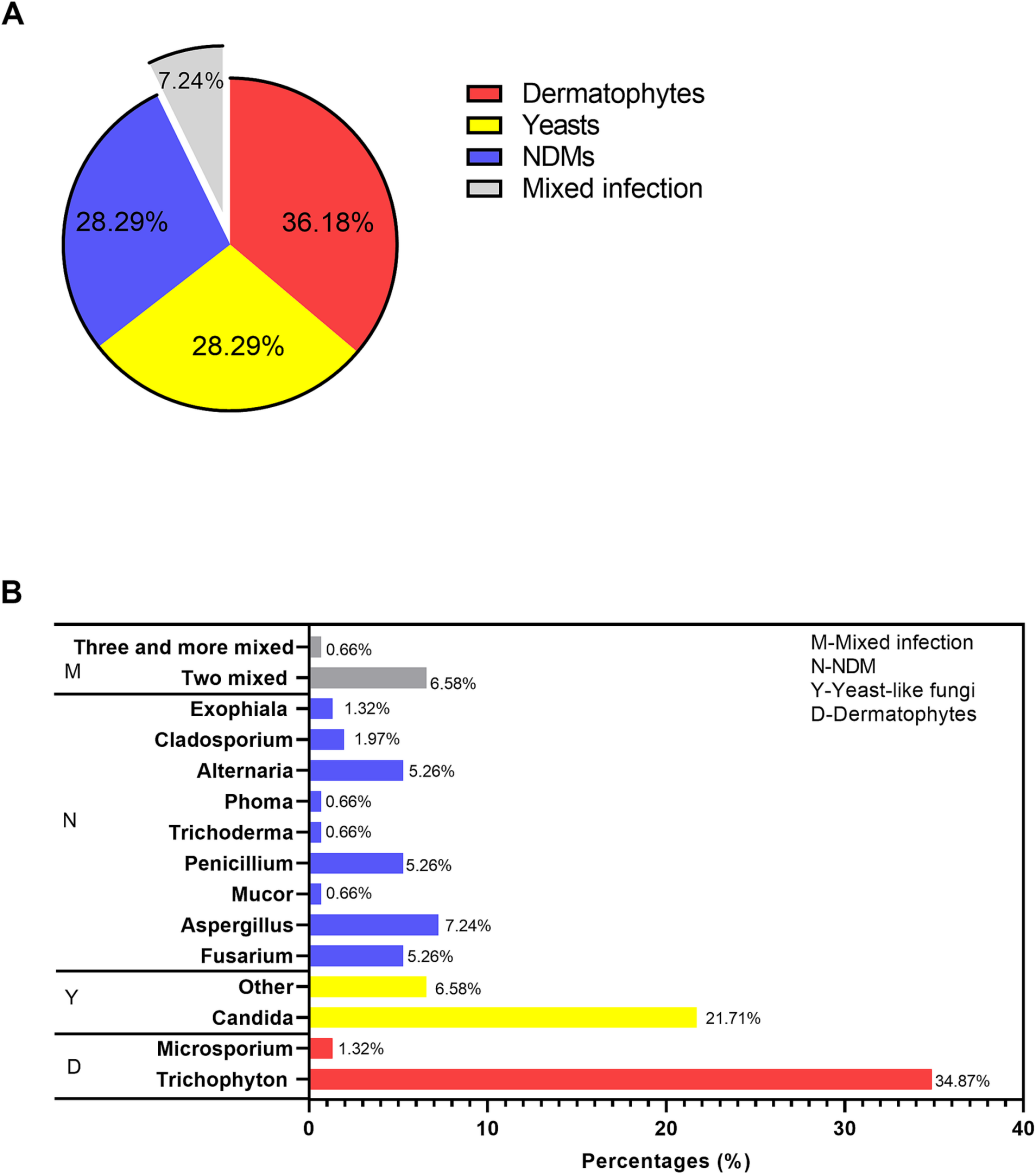
Causative agents in the fungal culture-positive patients suspected of onychomycosis. **(A)** The proportions of dermatophytes, yeast, NDMs, and mixed infections. **(B)** Genus distribution.

Among all the fungi causing onychomycosis (shown in Figure 2A), dermatophytes remained the predominant causative agent (36%), followed by yeast fungi (28.29%), non-dermatophyte molds (28.29%), and mixed infections (7.24%). Regarding the genus distribution (as shown in Figure 2B), *Aspergillus* (7.24%), *Fusarium* (5.26%), *Penicillium* (5.26%), and the dematiaceous fungi *Alternaria* (5.26%) were identified. At the species level, *Trichophyton rubrum* was the most commonly isolated dermatophyte (47/55), while *Candida parapsilsis* was the most commonly isolated yeast within the *Candida* genus (21/33), followed by *Candida albicans* (8/33) (Supplementary Table 2).

### 3.4 Different predominant causative fungi across gender and age

**Figure 3:**
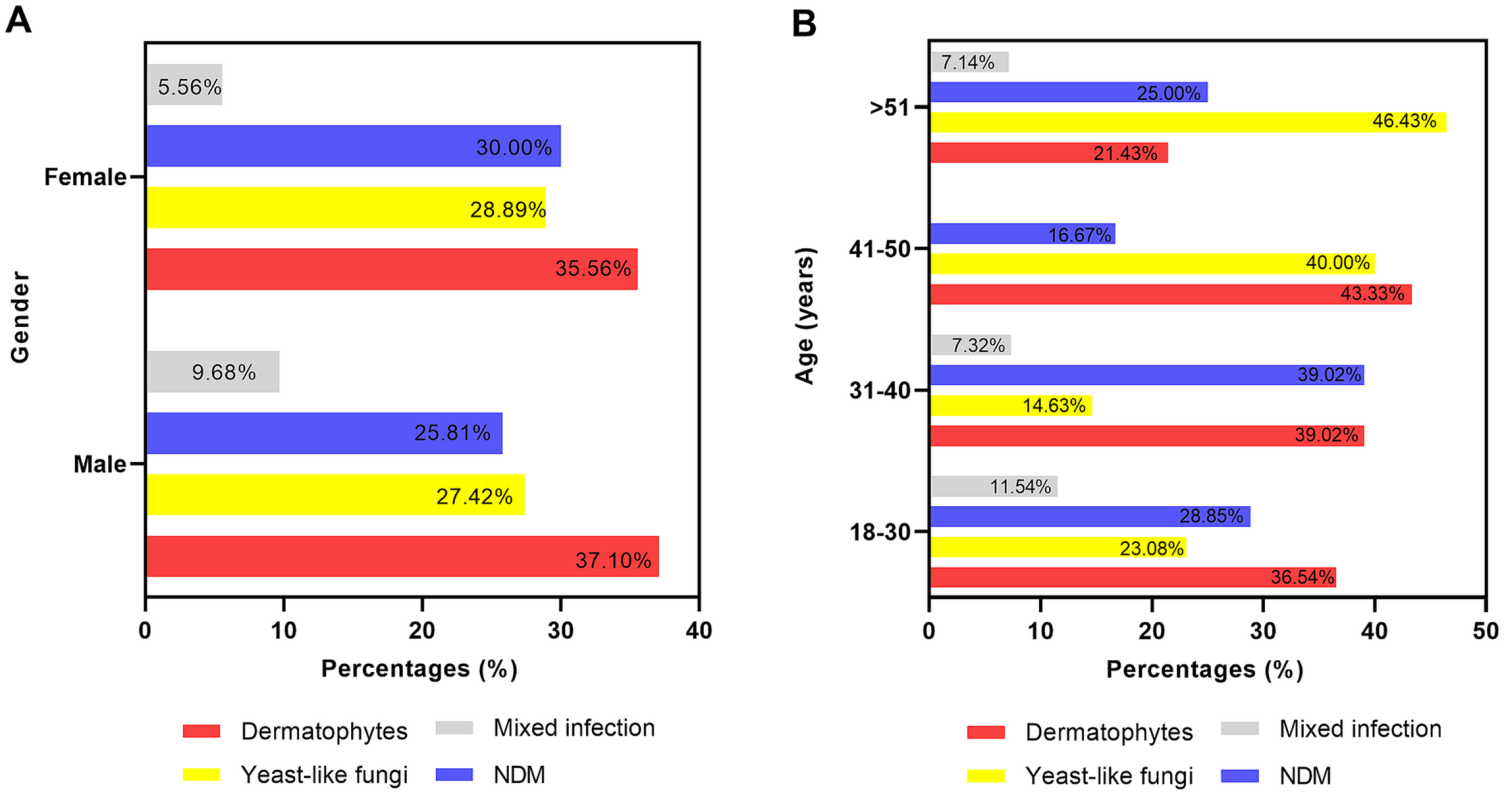
Different predominant causative fungi across gender **(A)** and age **(B)**.

In total, the number of samples with fungal growth was 62 in men and 90 in women. The distribution of the causative agents across different genders is shown in Figure 3A. Dermatophyte was the predominant causative agent in both men (*n* = 23) and women (*n* = 32) patients.

Regarding the profile of microbial agents across different age groups (shown in Figure 3B), dermatophytes were the most common fungi in the patients under 50 years of age (18–30: 36.54%; 31–40: 39.02%; 41–50: 43.33%, respectively). In the older patients over 51 years of age, yeast fungi were the most common (46.43%). NDMs were the second most common agents in most age groups (18–30, 28.85%; 31–40: 39.02%; >51:25%).

### 3.5 Distribution of different causative agents in the toenails and fingernails

**Figure 4:**
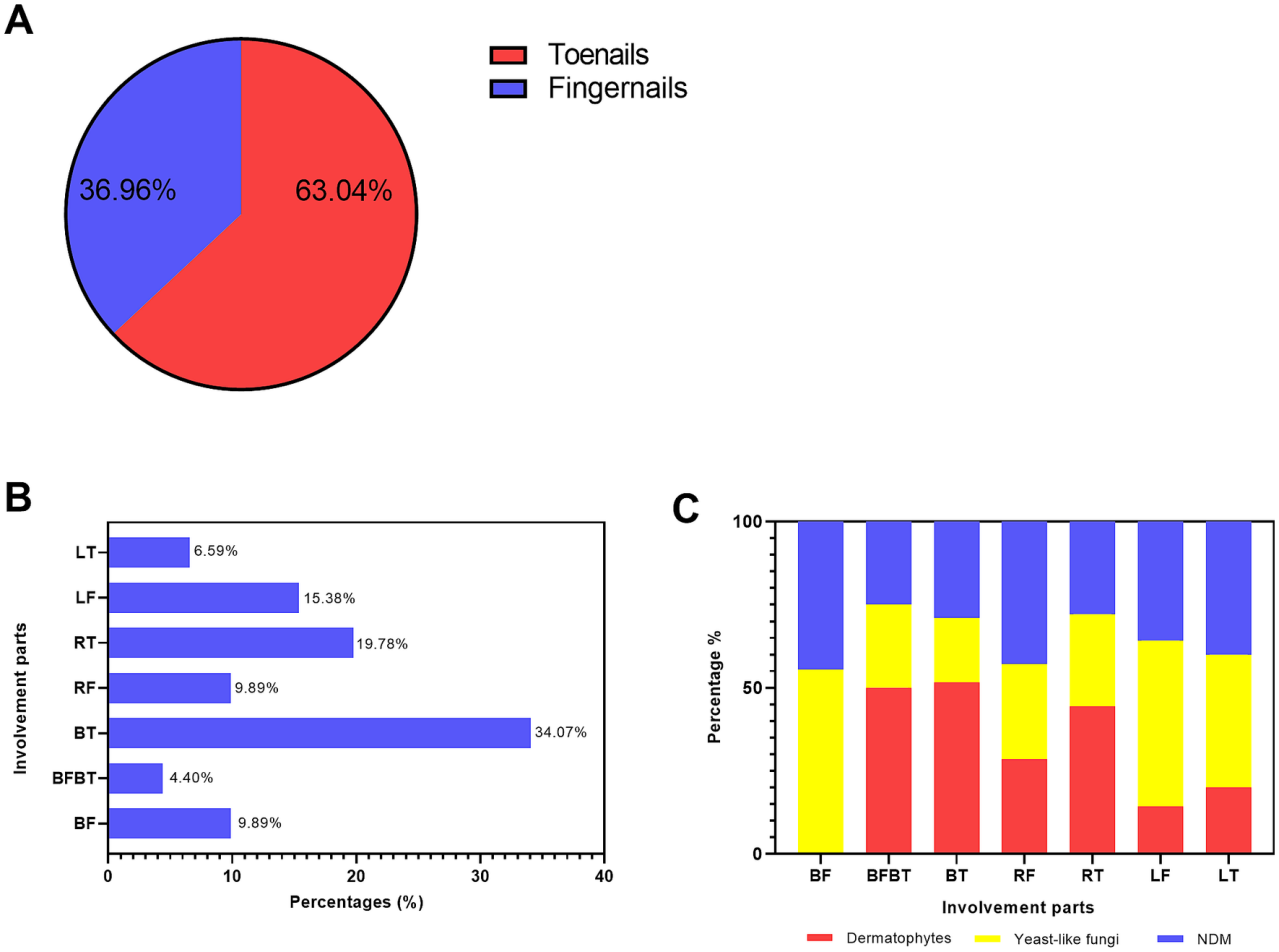
Distribution of different causative agents in the toenails and fingernails. **(A)** The proportions of toenail and fingernail involvement. **(B)** The percentages of different involvement types: LT, left toenail; LF, left fingernail; RT, right toenail; RF, right fingernail; BT, both left and right toenails; BFBT, both toenails and both fingernails; and BF, both fingernails. **(C)** The percentages of the three main causative agents—dermatophytes, yeast, and NDMs—in different involvement types.

First, we investigated the involvement of toenails and fingernails in onychomycosis. Due to the availability of detailed data, 91 patients were finally identified. In general (as shown in Figure 4A), fungal growth was more frequently observed in the toenail (63.04%) specimens than in the fingernail specimens (36.96%). Regarding the specific types of nail involvement (shown in Figure 4B), both left and right toenail involvement was the most common type (34.07%), followed by right toenail involvement (19.78) and left fingernail involvement (15.38%). Involvement of both toenails and fingernails was the least common type (4.40%).

Regarding the distribution of different fungi types in the toenails and fingernails (as shown in Figure 4C), mixed infections were recorded in only three patients and were therefore excluded from this comparison. Among the other three types of causative fungi, dermatophytes were the most predominant in both toenails and fingernails (50.0%), bilateral toenails (51.61%), and right toenails (44.44%). Yeast fungi were the most predominant in bilateral fingernails (55.56%), left fingernails (50.00%), and left toenails (40.00%).

## 4 Discussion

In this study, the number of samples sent for fungal culture identification (*n* = 298) did not match the number of outpatients clinically suspected of onychomycosis (*n* = 3,458) The turnaround time (TAT) for fungal culture identification usually takes approximately 2 weeks, which limits the acceptance of fungal culture tests by both patients and doctors—especially given the growing popularity and faster TAT of direct fungi fluorescence tests. However, the sensitivity of direct fungal fluorescence in onychomycosis is only 78% (Bao et al., 2018). This study may serve as a call for attention to the importance of fungal culture identification—not only for onychomycosis but also for other fungi infections.

In general, the culture-positive prevalence of onychomycosis varies across geographic locations, demographic features, and laboratory conditions, among other factors. In our study, the annual positive rate of fungal culture was approximately 80%, which is in line with a meta-analysis showing that fungal culture has a sensitivity of 29 ~ 82% in onychomycosis (Lim et al., 2021). Notably, a lower positive rate (50%) was observed in 2019, which may be partially due to the smaller number of samples sent for fungal culture that year. A prospective study by Reddy et al. showed a 66.66% culture-positive rate for onychomycosis in India (Reddy et al., 2012). David et al. reported a culture-positive prevalence of 33.1% for onychomycosis in a retrospective study conducted in Spain (Navarro-Pérez et al., 2023). In a smaller-scale study by Zhang et al., only 36% of fungal culture samples were positive among microscopy-positive samples (Zhang et al., 2012). Given the possibility of false negatives and the time-consuming nature of traditional methods, combined techniques such as molecular diagnostics may help improve clinical diagnostic accuracy.

Our results showed that fungi grew more frequently in the samples isolated from women. This is consistent with current views that the distribution of pathogens in onychomycosis is influenced by gender (Song et al., 2022a). In addition, since women are more likely to wear nail polish than men, nail polish may cause both physical injury and create a moist environment, which are the risk factors for onychomycosis (Lipner and Scher, 2019). Fungi grew more frequently in the samples obtained from young adults (age 18–30), which is consistent with the latest report on the peak age (between 16 and 45 years) of incidence in tinea pedis (Leung et al., 2023). However, it should be noted that aging has long been considered a risk factor for onychomycosis (Albucker et al., 2023; Perea et al., 2000). The higher rate of fungal culture positivity in young adults may be associated with their greater willingness to seek professional help compared to older patients. Therefore, future research with a larger sample size should be conducted to address this in northwest China.

The spectrum of fungal types differs depending on geographic location. In the current study, dermatophytes remained the predominant causative agent in onychomycosis, accounting for less than 50% (36.59%) in northwest China. In contrast, a recent study by Song et al. reported that dermatophytes accounted for more than half (60.59%) of cases in mainland China (Song et al., 2022a). This may be attributed to the weather and geographic features of Shaanxi Province, where temperatures and humidity are lower compared to coastal cities. Dermatophytes are much more infectious in hot and humid conditions (Parvez et al., 2023). Notably, the frequency of molds, other than dermatophytes, increased fourfold to 28%, compared to recent research in mainland China, where molds (7.91%) were the third most common causative agent (Song et al., 2022a). We reported that yeast fungi were the most common agent in the older patients aged over 51. The age-related distribution of yeast fungi is similar to that found in a previous study by Araiza-Santibánez et al. (2016), which identified yeast to be the second prominent fungi in older patients with onychomycosis. The distribution of yeast fungi in older patients may be due to a weakened immune system and comorbidities such as diabetes, which can hamper local immunity and increase susceptibility to yeast infections (Oliveira et al., 2023). The use of medications, such as immunosuppressive agents, can also disrupt the local microbe balance in older patients.

In this retrospective study, *Trichophyton rubrum* was the most isolated dermatophyte, which is in line with a recent 30-year nationwide study in China (Song et al., 2022b). However, unlike the result showing *Candida albicans* as the most isolated yeast in China (Song et al., 2022b) and most parts of the world (Paškevičius et al., 2020; Otašević et al., 2016), we reported *C. parapsilsis* as the most isolated yeast species, with more than twice the incidence of *C. albicans* in Shaanxi province. The distribution of fungi species is affected by complex factors. Similar to our study, Feng et al. reported *C. parapsilsis* as the most common species, followed by *C. albicans* in Shanghai, located in the East China region (Feng et al., 2015). Given the differences in antifungal susceptibility among these *Candida* species and the emergence of resistance, it is of great importance to monitor the pathogenic yeast profile and perform standard antifungal tests.

Regarding specific fungal genera, in addition to the commonly recognized fungi *Trichophyton* and *Candida*, it is important to be aware of the increasing rate of NDMs. All NDMs reported in this study were previously isolated in China and other parts of the world from patients with onychomycosis (Song et al., 2022a; Leelavathi et al., 2012). We found *Aspergillus* to be the most isolated NDM, followed by *Fusarium*, *Penicillium*, and the dematiaceous fungi *Alternaria*. This is consistent with a recent analysis by Song et al., who reported *Aspergillus* as the most predominant NDM, followed by *Penicillium* and Dematiaceae (which includes *Alternaria*, *Fonsecaea*, *Acremonium*, and *Scytalidium*) (Song et al., 2022a). Although NMDs were previously thought to be common contaminant organisms in the lab, their pathogeny in onychomycosis has also been demonstrated (Moreno and Arenas, 2010; Bongomin et al., 2018). Moreover, NDMs, including dematiaceous fungi, are the second most common causative agents. Dematiaceous fungi are a group of melanized fungi that can cause both cutaneous and systemic infections, especially in immunocompromised patients with mortality rates of up to 60% (Wu et al., 2016; Derber et al., 2010). Given the possibility of contamination in lab cultures, more attention should be paid and more repeated samples should be sent for testing when NMDs are reported.

Regarding the involvement of nails in onychomycosis, infection of both left and right toenails is the most common type (34.07%), followed by right toenail infection (19.78%) and left fingernail (19.78%) infection. Involvement of both toenails and fingernails is the least common type (4.40%). Overall, the toenails were much more commonly involved compared to the fingernails in our study, which is in line with other reports, whether hospital-based or population-based (Sigurgeirsson and Baran, 2014; Khalifa et al., 2023). The slow growth of toenails, reduced blood supply, and a moist environment may partially account for the higher susceptibility of the toenail to onychomycosis (Westerberg and Voyack, 2013).

There are some limitations in the current study. First, in northwest China, since onychomycosis has long been neglected in research, the number of patients suspected of onychomycosis did not match the number of samples sent for fungal culture identification. Second, the pathogenic role of NDMs in onychomycosis needs further investigation, including their virulence and susceptibility to antifungal tests. Third, this study was a single-center, hospital-based study, which might have weakened the strength of the analysis of the demographic data. Finally, the number of culture-positive samples was relatively small, so the fungal profile distribution should be interpreted with caution. Moreover, in the future, studies with larger sample sizes should be conducted to validate the results.

## 5 Conclusion

Despite these limitations, to the best of our knowledge, this is the first analysis of the causative agents of onychomycosis in northwest China, a region where onychomycosis has been neglected in research for a long time. The present study highlights that dematiaceous fungi causing onychomycosis may be an emerging concern in northwest China. Moreover, this study serves as a call to raise awareness about the importance of fungal culture, especially in the context of the increasing presence of dematiaceous fungi.

## Data Availability

The original contributions presented in the study are included in the article/Supplementary material, further inquiries can be directed to the corresponding authors.
