# Supplementary material for: A six-year retrospective study on the causative agents of onychomycosis in China: the emergence of dematiaceous fungi
Source: Front Microbiol. 2025 May 1;16:1582147. doi: 10.3389/fmicb.2025.1582147 (PMC12078243; doi:10.3389/fmicb.2025.1582147)
Supplement: Supplementary file 1 [file Data_Sheet_1.docx]

Supplementary files

For

A six-year retrospective study on the causative agent of onychomycosis in China: Emerging dematiaceous fungi

Supplementary Table 1 Clinical demographic features of patients suspected of onychomycosis

| **Factors** | | **Number** |
| --- | --- | --- |
| **Comorbidities** | Tinea cruris | 1 |
|  | Chronic Paronychia | 2 |
|  | Tinea pedis | 39 |
|  | Ingrown Toenail | 3 |
|  | tinea manuum and pedis | 3 |
|  | NA | 104 |
| **Underlying diseases** | Hypertension | 4 |
|  | Diabetes mellitus | 8 |
|  | Coronary Heart Disease | 2 |
|  | Hypothyroidism | 2 |
|  | Chronic gastritis | 2 |
|  | Cerebral infarction | 7 |
|  | Hyperlipidemia | 1 |
|  | NA | 132 |
| **History of antifungal use** | Topical medication | 80 |
|  | Systemic medication | 6 |
|  | NA | 66 |
| **Occupational exposure** | nail cosmetology | 1 |
|  | Foot bath massage therapist | 1 |
|  | NA | 150 |
| **History of nail trauma** | Yes | 12 |
|  | NA | 140 |

Supplementary Table 2 Fungi species distribution

| **Fungi** | | **Number** |
| --- | --- | --- |
| **Dermatophytes** | Trichophyton rubrum | 47 |
|  | Trichophyton mentagrophytes | 2 |
|  | Trichophyton tonsurans | 1 |
|  | Other Trichophyton | 3 |
|  | Microsporium genus | 2 |
| **Candida Spp** | Candida albicans | 8 |
|  | Candida glabrata | 1 |
|  | Candida parapsilsis | 21 |
|  | Candida krusei | 2 |
|  | Candida tropicalis | 1 |
